# Nucleic acid base pair open states by hydrogen exchange

**DOI:** 10.1073/pnas.2520855122

**Published:** 2025-12-30

**Authors:** S. Walter Englander

**Affiliations:** ^a^Department of Biochemistry and Biophysics, Perelman School of Medicine at the University of Pennsylvania, Philadelphia, PA 19104

**Keywords:** hydrogen exchange, DNA dynamics, base pair opening, solitons

## Abstract

Nucleic acid base pair open states that are actively driven to expose normally hidden sequences and host reactions with processing proteins have been highly studied. However, the spontaneous natural base pair opening behavior of native duplexes is still not understood. Hydrogen exchange (HX) experiments can measure the kinetic and equilibrium parameters of base pair opening reactions, but different approaches have led to discrepant datasets and opposing views of nucleic acid structural dynamics. Here, I reexamine the data and resolve these long-standing contradictory views. The conclusion is that the seemingly contradictory datasets characterize two different modes of base pair opening reactions that, for identifiable reasons, are selectively accessed by the different methods and molecular models used. H-T exchange of DNA, duplex RNA, and long synthetic duplexes is dominated by large multi-base-pair openings with reclosing times of milliseconds (kcl ~20/sec) and surprisingly high population (Kop ~10^−2^). H-H exchange measured by NMR relaxation is limited to the study of small rapidly tumbling oligonucleotides. Their HX is dominated by single base pair openings with reclosing times of microseconds and extremely low population (Kop ~10^−6^). The different openings are seen selectively by the different methods because small oligonucleotides cannot host the extensive openings, while in large polynucleotides, the minimal population of single base pair openings makes no perceptible contribution to measured HX. The unexpected kinetics, equilibrium occupation, and size of DNA base pair openings are suggestive of rapidly migrating traveling loop wave packets known as solitons that dynamically scan and expose candidate recognition sites.

## Significance

Hydrogen exchange (HX) experimentation has made major contributions to the understanding of dynamics and function in proteins, but the same is not true for nucleic acids. This has been due to a misunderstanding of the correct interpretation of nucleic acid HX behavior in terms of nucleic acid dynamics. This paper reexamines available data, develops the interpretation, and resolves a long-standing fundamental conflict. The analysis also explains the bases of a proposed soliton hypothesis, its application to DNA HX behavior and molecular dynamics, and possibly to DNA–protein and DNA–DNA interactions.

The study of nucleic acid hydrogen exchange (HX) has long been motivated by the puzzle of how proteins that modulate gene expression manage to recognize and interact with target nucleotide sequences that are structurally hidden inside the DNA double helix. Nucleic acids under native conditions experience spontaneous base pair opening reactions that expose normally hidden nucleotide sequences and may mediate specific interactions that control replication, transcription, restriction, and so on. The base pair opening and reclosing kinetics and equilibria that govern these interactions can be measured by HX methods. Problematically, the two major HX methods that have been used in nucleic acid HX studies, the H-T exchange labeling method (1) and NMR H-H exchange methods (2), have found sharply different HX parameter sets with very different implications for DNA physical chemistry, dynamics, and function. The conflict, which dates back to the 1990s and earlier, has never been resolved.

**Fig. 1.**
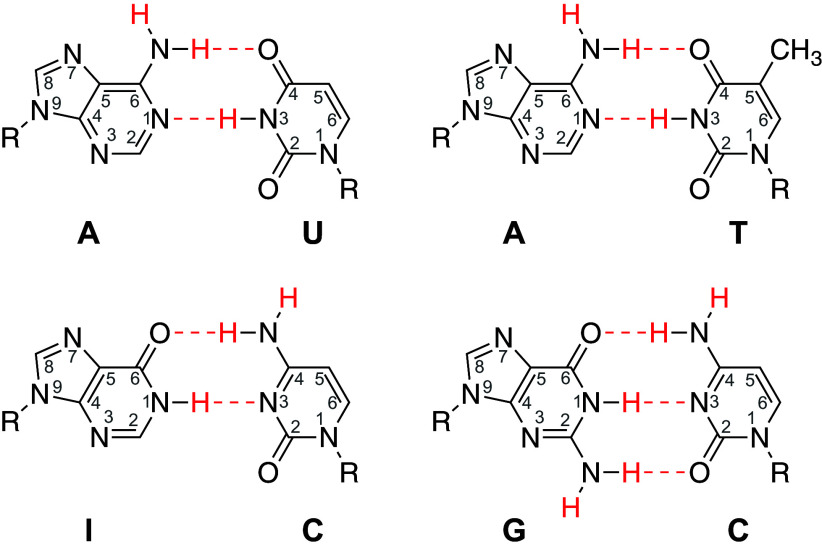
Nucleic acid base pairs with their exchangeable protons in red. All H-exchange proceeds from a base pair transiently open state. The imino protons although fully buried in the duplex are the fastest to exchange. When base pair reclosing is slower than imino proton exchange from the open state, its HX rate reveals the base pair opening rate (kop). The paired amino group protons, with one proton internally H-bonded and one exposed to solvent, both exchange with solvent protons at the same rate, which reveals the base pair equilibrium opening constant (Kop) and the base pair reclosing rate (kcl).

Fig. 1 pictures the chemical structure of Watson–Crick H-bonded nucleic acid base pairs. The nucleotides have two kinds of exchangeable protons, the endocyclic imino proton and the exocyclic amino group protons. In duplex nucleic acids, the ring imino proton at N3 of U and T N1 of I and G, and one of the two exocyclic NH2 amino protons on A, C, and G are buried and H-bonded. Yet they all engage in continuous exchange with solvent water protons. The HX rates of the imino and amino protons depend on and therefore encode quantitative information on the kinetics and thermodynamics of base pair opening behavior. Eliciting that information requires an understanding of HX and proton transfer theory. The present analysis summarizes the theory and relevant data and reexamines and resolves the outstanding conflict. The paper then considers a soliton hypothesis that may explain the HX data in terms of a trapped traveling wave dynamic that rapidly scans through DNA, exposing a changing set of candidate nucleotide sequences that can foster specific DNA interactions.

## Theory

### HX (Linderstrøm–Lang Theory).

Following the discovery by Linus Pauling of the role of H-bonding in helices and sheets (3, 4), Kai Linderstrøm–Lang inferred (5, 6) that structurally slowed HX depends on stable H-bonding and that the exchange process requires the prior dynamic opening of the protecting H-bond to expose the hydrogen to solvent catalyst acceptors, as in Eqs. **1**–**4**. Measured HX rates can then reveal kinetic (kop, kcl) and equilibrium (Kop) structural opening parameters.

[1] $$\mathrm{Closed}\overset{\mathrm{kop}}{\underset{\mathrm{kcl}}{↔}}\mathrm{Opened}\overset{\mathrm{kch}\left[\mathrm{Cat}\right]}{\to }\mathrm{Exchanged}:\mathrm{Kop}=\mathrm{kop}/\mathrm{kcl},$$

[2] $$\begin{array}{c} \mathrm{kex}=\mathrm{kop}\;\times \;\mathrm{kch}\left[\mathrm{Cat}\right]/\left(\mathrm{kop}+\mathrm{kch}\left[\mathrm{Cat}\right]+\mathrm{kcl}\right), \end{array}$$

[3] $$\mathrm{EX}1:\mathrm{kch}\left[\mathrm{Cat}\right]>>\mathrm{kcl}:\mathrm{kex}=\mathrm{kop},$$

[4] $$\mathrm{EX}2:\mathrm{kch}\left[\mathrm{Cat}\right]<<\mathrm{kcl}:\mathrm{kex}=\mathrm{Kop}\;\times \;\mathrm{kch}\left[\mathrm{Cat}\right].$$

The HX rate measured for a dynamically exposed proton depends on a kinetic competition between the catalyzed exchange rate and base pair reclosing (Eqs. **1** and **2**). Upon base pair opening, transfer of the proton to OH^−^ or to another solvent catalyst may be faster than base pair reclosing. In this case, measured exchange will be opening-limited and appear to be catalyst-independent, revealing the base pair opening rate (Eq. **3**). This is called EX1 (monomolecular) exchange (Eq. **3**) (7). If reclosing is faster than the exchange reaction, opening and reclosing will occur repeatedly before exchange is successful. In this EX2 (bimolecular) situation, the measured exchange rate proportions to the fraction of time open and it reveals Kop (Eq. **4**).

### Proton Transfer (Eigen Theory).

Once exposed to solvent by H-bond separation, proton removal can be catalyzed by encounter with a solvent base at a rate dictated by the rules enunciated in the proton transfer theory of Manfried Eigen (kch[Cat] in Eq. **1**) (8). Upon fast diffusion-limited collision and immediate H-bond formation to a catalyzing acceptor (Cat), the proton distributes to equilibrium across the H-bond as determined by the relative basicities of the donor and the acceptor. For the nucleotide imino proton donor (pK ~ 9) and hydroxide acceptor (pK ~ 16), the equilibrium within the encounter complex is far toward hydroxide, by a factor of 10^7^ (10^∆pK^). The proton always leaves with the catalyst. Every collision is successful. The HX rate is limited by and defines the base pair opening rate (kop).

For the amino protons, the reverse is true. Nucleotide amino groups are much weaker acids (donor) than the imino protons, with pK about 20. In the collision complex, the proton equilibrium is far toward the amino donor, and many opening/closing and collision events must occur before a successful transfer. This is called an EX2 (bimolecular) HX reaction (Eq. **4**). Direct collision-dependent exchange is so slow that a different two-step reaction pathway dominates (9, 10) the exchange process. A first step protonates the transiently exposed ring N (pK ~ 4), which lowers the amino group’s pK and promotes the catalytic removal of its protons. Still, exchange can occur only after multiple openings and reclosings, and it can be accelerated by added solvent catalyst. The acceptor may be either the strong base OH^−^ or a weaker solvent base with lower pK although at much higher concentration. Exchange will then appear to be general acid catalyzed. The HX slowing factor, the measured rate divided by the known faster rate for the fully open and unhindered situation, gives the fraction of time open, the equilibrium constant for base pair opening (Kop) (Eq. **4**).

Lang’s view of HX kinetics dependent on structural dynamics and Eigen’s chemical proton transfer theory have both been thoroughly substantiated and, after a long period of uncertainty (11), now informs all HX analysis.

## Results

This section presents illustrative HX results taken from the literature that are typical of a quantity of data obtained using the methods and molecular models discussed. A proper interpretation of the data defines the kinetic and equilibrium parameters of base pair open states, resolves the long-standing conflict between the different approaches that have been used to study nucleic acid HX, and leads to the DNA soliton hypothesis.

### H-T Exchange of Polynucleotides.

We used our tritium exchange methods (H-T exchange) (1) to study the HX behavior of ribo and deoxyribo homo and alternating synthetic polynucleotides of A-U, A-T, I-C, and G-C pairs (12, 13) with polymer lengths on the order of 1,000 base pairs, as well as tRNA (14) and chromatin (15). Peter von Hippel and his students used the same methods to examine DNA itself (9, 16, 17). Many others subsequently applied developing high-resolution NMR methods to measure H-H exchange in a variety of short oligonucleotides at base pair resolution. Here, I illustrate and reconsider the interpretation of that data.

**Fig. 2.**
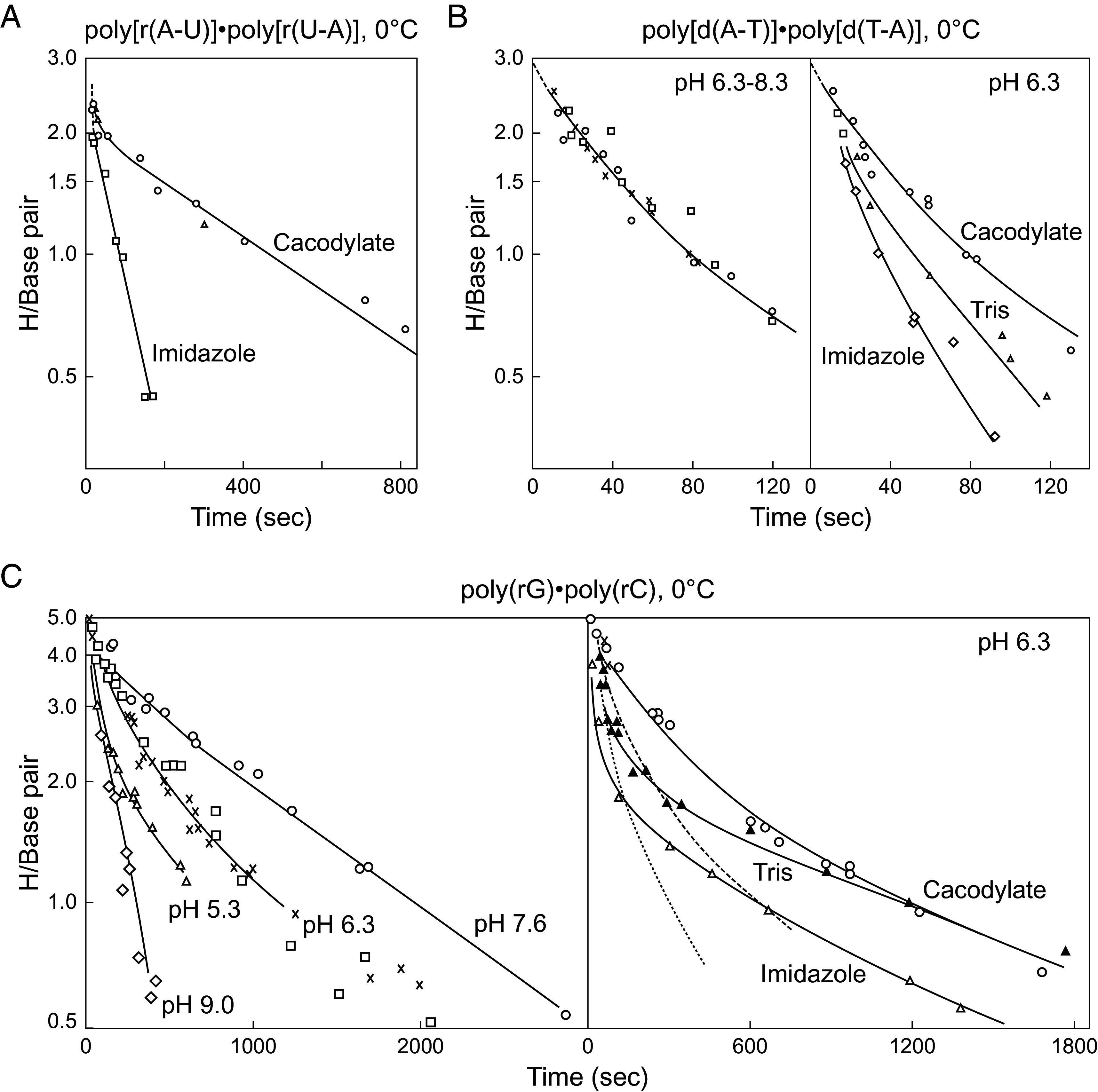
H-T exchange of several polynucleotides. (*A*, *B*) All of the Watson–Crick H-bonded hydrogens are measurably slow. Exchange of the faster proton is not catalyzable, indicating opening-limited exchange (EX1). This is the imino proton. The slower pair of amino group protons (two pairs in G-C) are distinguished by their catalyzability by OH^−^ -ion and small molecule bases (EX2). Various catalyst concentrations were used to set a conveniently measurable HX time scale in each case. Dotted curves in panel *C* multiply the faster part of the cacodylate curve for comparison with the catalyzability of the two different pairs of G-C amino protons. The faster G-C pair matches the response to Tris of the cytosine amino protons in poly(rI).poly(rC) (12), relegating the slower pair to the guanine amino group. Reprinted from ref. 12 with permission from Elsevier.

**Fig. 3.**
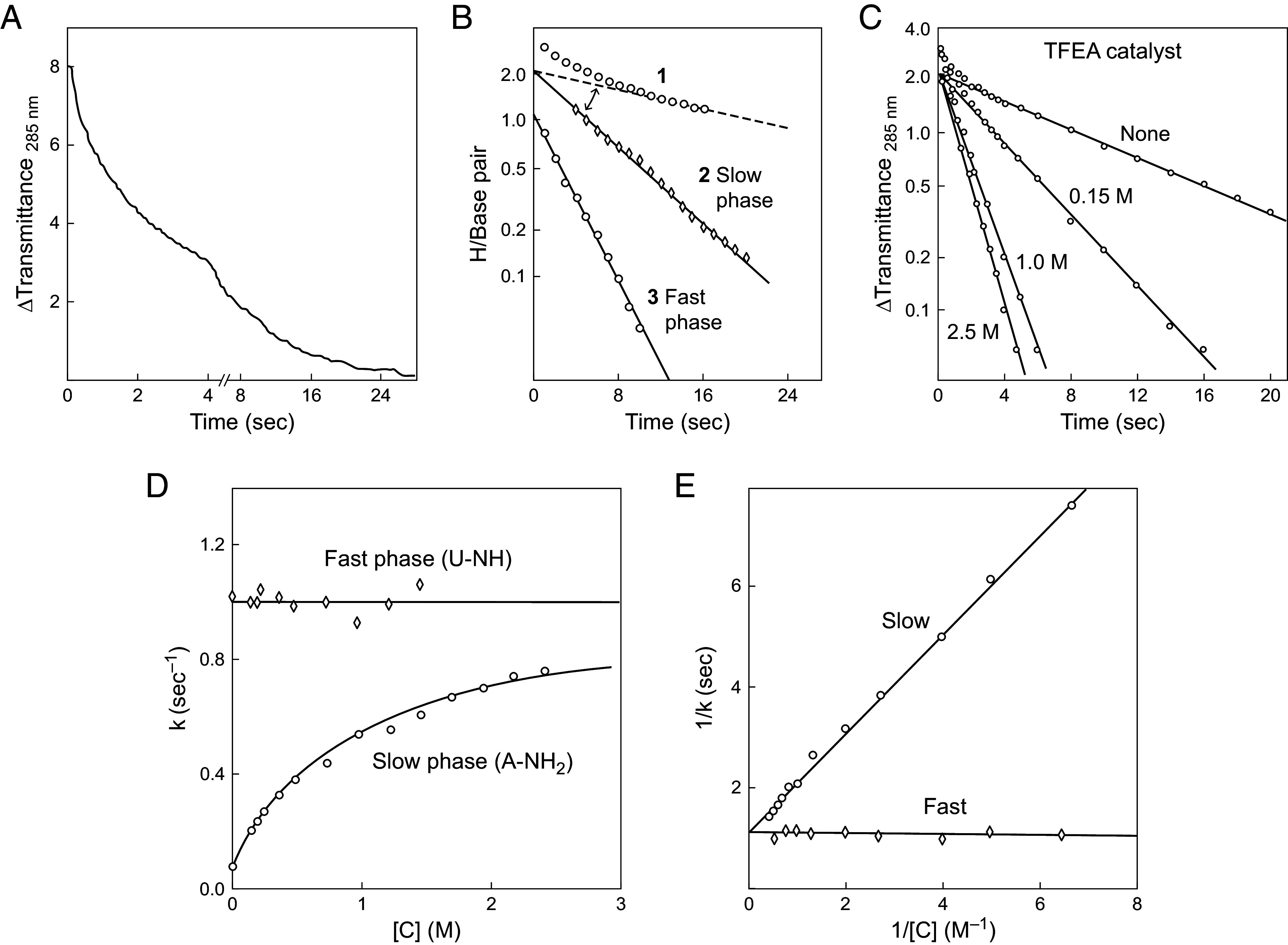
Complete HX analysis for poly(rA).poly(rU) (20 °C, pH 7) (18). (*A*) The fully deuterated polymer was mixed into H_2_O by stopped-flow and D to H exchange was measured by change in transmittance at 285 nm. (*B*) Semi-log plots of the slow and fast phases. (*A* and *B*) Reprinted from ref. 18 with permission from Elsevier. (*C*) The two slow-phase adenine amino protons are equally catalyzed by added trifluoroethylamine (TFEA) because they both exchange from the open state and respond to the same two-step reaction pathway. The fast phase imino proton is not catalyzed because its exchange is opening-limited. (*D*) Fast and slow-phase HX rates versus TFEA concentration. (*E*) Double inverse plot of HX rates vs. catalyst concentration. At high catalyst concentration, the slow-phase NH2 rate asymptotes to the fast phase opening-limited imino rate, demonstrating that both exchange from the same open state. (*C*–*E*) Reproduced with permission from ref. 11.

Figs. 2 and 3 show a sampling of H-T exchange results for various synthetic polynucleotides. The HX of all of the exchangeable protons and not just the Watson–Crick H-bonded protons is slow enough to be measurable on the time scale of seconds and longer. In all cases, the HX data distinguish one very fast exchanging proton per base pair and one pair (A-U, A-T, I-C) or two pairs (G-C) of slower protons.

When measured by H-T exchange, alternating polymers are more stable (slower HX) than homopolymers, ribopolymers are more stable than deoxy, and G-C polymers are more stable than A-U and A-T, all as expected from known whole molecule cooperative DNA properties. NMR studies of H-H exchange in short oligonucleotides have sometimes revealed opposite sensitivity (19), apparently because they measure a more local base pair opening process (see below.)

HX of the single fast proton per base pair is pH-independent and insensitive to general catalysis (EX1). It represents the ring imino proton. When exposed by base pair opening, imino protons with pK ~ 9 exchange on first contact with OH^−^ catalyst in milliseconds or even faster to buffer base. Thus, exchange is faster than reclosing, it displays opening-limited EX1 exchange, and it reveals the base pair opening rate (kop) (Eq. **3**). The opposite behavior in oligonucleotides, producing catalyzable EX2 exchange, is discussed below.

The complex HX reaction kinetics of adenine amino protons, defined in stop flow and NMR studies of AMP and polyrA (10, 20), serves to identify the adenine amino group protons. The amino group has high basicity. Its exchange depends on lowering its pK indirectly by an initial protonation on the ring N1. Exocyclic amino group pK then goes from ~20 to ~9, promoting facile proton transfer to OH^−^ and other catalyzing buffer base acceptors. Similar pathways although with differing parameters obtain for the cytosine amino group protons and the two amino groups of the G-C base pair.

Both protons in each NH2 proton pair exchange at the same EX2 rate and they are equally catalyzed by general bases, even though one is solvent-exposed and one is internally H-bonded. This occurs because HX of both protons of the NH2 pair follows the preprotonation of a normally buried but transiently exposed ring N so that both amino protons exchange from the base pair open state. The EX2 exchange of the amino protons, when compared with the rate for the free nucleotide (e.g. AMP), reveals the fraction of time the amino protons are available for exchange, equal to Kop, the base pair equilibrium opening constant (Eq. **4**).

Fig. 3 shows a detailed analysis of poly(rA).poly(rU) HX behavior. The H-D exchange data were collected by stopped-flow monitored by optical absorbance to accurately measure the fast and slow phases (Fig. 3*A*). As before, the single fast phase imino proton of uracil exchanges in an EX1 manner. It is opening-limited, not catalyzable (Fig. 3 *C* and *D*), and it reveals the base pair opening rate (kop) (Eq. **3**). The two slow-phase adenine amino protons exchange at the same slower rate, are equally catalyzed by specific and general base, and they reveal the base pair equilibrium opening constant. A double inverse plot of the HX data (Fig. 3*E*) shows that the slow amino pair, when it is strongly catalyzed, accurately asymptotes to the opening-limited imino proton rate (derived from Eq. **2**).

This is precisely the behavior expected for the Linderstrøm–Lang model when both kinetic phases, the fast opening-limited imino proton EX1 phase and the slower catalyzable amino proton EX2 phase, are determined by the same opening reaction. This important result can be taken to validate the Lang model and the interpretation of these HX data.

### H-H Exchange of Oligonucleotides by NMR.

**Fig. 4.**
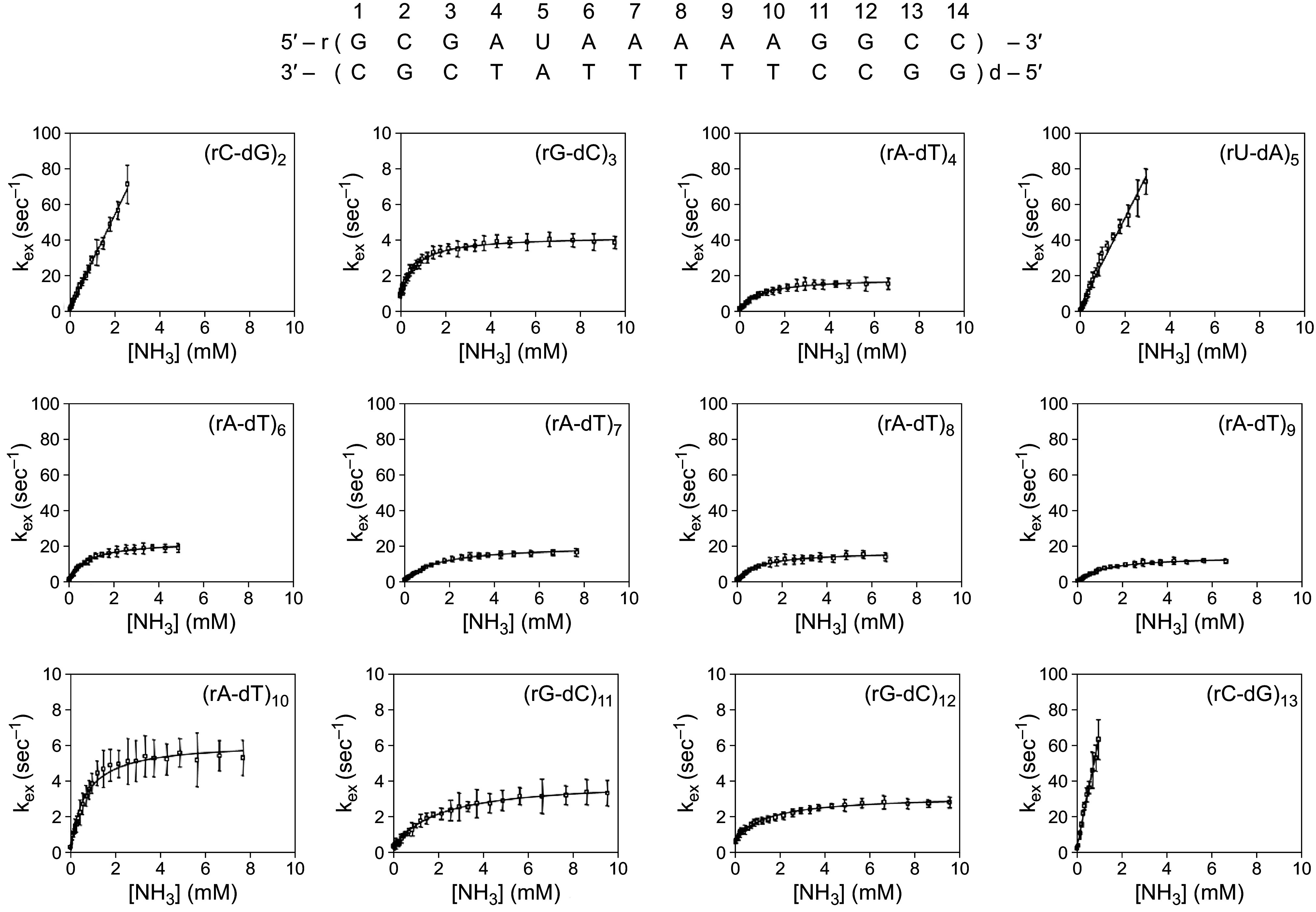
HX of the individual imino protons in a short r/d hybrid oligonucleotide with catalysis by NH3. The oligomer is stabilized against end fraying by several terminal G-C pairs. Individual imino proton resonances were assigned and their HX behavior was measured by water inversion-recovery NMR (10 °C) (21). Reprinted with permission from ref. 21. Copyright 2017 American Chemical Society.

Just as nucleic acids provided an early target for H-T exchange experiments, early developments in high-field NMR turned to nucleic acid HX studies. At the time, NMR was limited to the study of small oligonucleotides which tumble rapidly (2) and to the low-field imino protons. The amino protons resonate at even lower field making their measurement difficult. Fig. 4 shows H-H exchange NMR results for a small 14 base pair r/d hybrid duplex (21, 22), measured by the HX-dependent T1 relaxation of solvent water. The focus on water relaxation avoided the problems inherent in measuring relaxation of protons of the duplex itself (23). These results are typical for many NMR studies of naturally occurring and synthetic short ribo and deoxyribo oligonucleotides (2, 24, 25).

The catalyzed HX rate for the imino proton in each base pair reaches a plateau indicating the opening-limited HX rate (kop). Measured kop is similar to the value found by H-T exchange for large polynucleotides and initially suggested that these different molecules exchanged similarly. However, more complete results have found large differences in other parameters. Also, the NMR experiment finds that the imino proton, far from being opening limited, is catalyzable (Fig. 4).

Closing rates (kcl) and equilibrium fraction open (Kop) can be calculated from the HX rates in the catalyzed region since HX here is in the EX2 regime. These values, listed in Table 1, display the striking difference in base pair kcl and Kop parameters determined by H-T exchange and NMR. They are different by 1,000-fold. The differences are not a function of nucleotide sequence effects. For example, compare the values found by H-T exchange for the long A-U and A-T runs in the homopolymers with the short oligonucleotide A-T runs measured by NMR (Table 1).

**Table 1.** Kinetic and equilibrium parameters of base pair opening reactions

H-TX	Kop	kop/s	kcl/s	T °C
polydA.polydT	0.05	2	40	25
polyrA.polyrU	0.01	0.06	6	0
polyrA.polyrU	0.05	1	20	20
polyrI.polyrC	0.004	0.1	25	0
polyrG.polyrC	0.0004	0.01	25	0
DNA	0.005	0.04	10	0
**H-HX by NMR**				
14-mer rA-rU	10^−5^	50	10^7^	10
14-mer dA-dT	10^−6^	5	10^7^	10
14-mer r/d hybrid	10^−5^	10	10^6^	10

Parameters shown were obtained from H-T exchange of large polymers or from H-H exchange measured by NMR for short oligomers. H-T exchange values are sensitive to the stability of long nucleotide sequences. This is much less so for the NMR results. Note the contrast in Kop and in kcl between large polymers and short oligomers. H-T results are from refs. 12, 13, 18, and 26. DNA results are from refs. 17 and 27. NMR results are from refs. 19 and 21.

### Comparison with DNA.

**Fig. 5.**
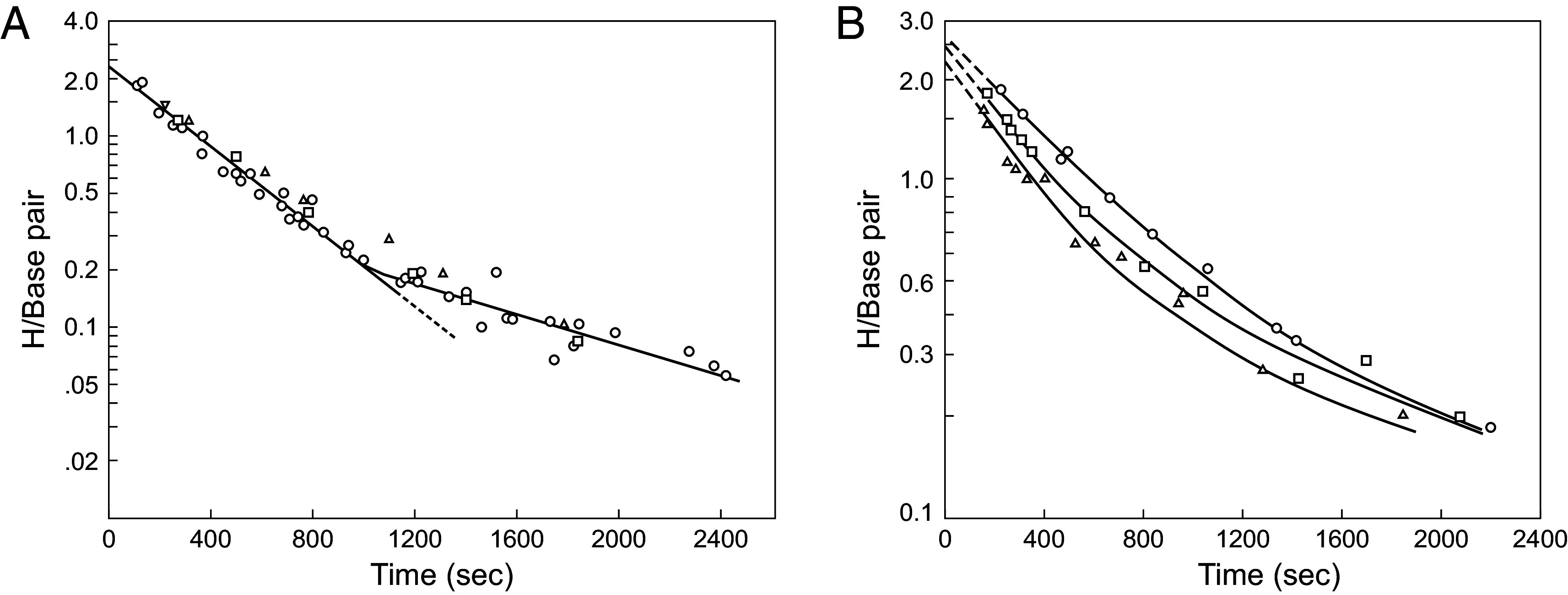
DNA H-T exchange (9, 16, 17, 27). (*A*) Calf thymus DNA (3.5 °C, pH 7.6; sonicated and unsonicated). HX rate of the amino protons, which reflect stability, varies by less than threefold through the entire exchange curve. (*B*) Native DNAs (0 °C) with G-C base pair content of 31%, 42%, and 72%. HX rates vary by only about twofold. Exchange times measured are not early enough to show the fast exchanging imino protons. Reprinted with permission from ref. 16.

Fig. 5 reproduces H-T exchange results for DNA obtained by Printz and von Hippel (27, 28).

In spite of the large difference in HX rate between homogeneous stretches of poly(A-T) and poly(G-C) (Fig. 2 and Table 1), HX of the amino protons of DNA shows little spread in HX rates (Fig. 5*A*). Even short runs of A-T or G-C sequences can be expected to contribute particularly fast or slow segments, but this is not seen. Further, DNAs with very different G-C content, and thus very different stability, show similar rates (Fig. 5*B*). These results indicate that the operative HX opening reactions do not vary simply with immediate local stability. Rather, I suggest, they reflect a multinucleotide opening that averages the stabilities of a sizable number of base pairs that open together (9, 16, 17).

### Summary of Results.

Table 1 summarizes available information on kinetic and equilibrium parameters of base pair opening reactions in duplex polynucleotides.

## Discussion

This paper reconsiders the present understanding of an important aspect of DNA physical chemistry, its naturally occurring base pair separation or “opening” behavior. This behavior represents a fundamental step in DNA structural dynamics and, it seems likely, in DNA–protein and DNA–oligonucleotide interactions. The results gathered in Table 1 summarize the essential content of HX experimentation that began soon after the original development of HX studies (5), continued for many decades thereafter (2), and led to conflicting results that have never been satisfactorily explained.

### Controversy and Resolution.

Early NMR studies of short oligonucleotides found opening-limited imino proton HX rates close to those found before for polynucleotide H-T exchange, suggesting that the same opening reactions were being measured by both methods. More advanced results point to sharp contradictions (Table 1). NMR finds the base pair reclosing rate to be very fast, ~10^6^ per sec. This makes the NMR-detected open state lifetime short and its equilibrium occupation small (Keq ~ 10^−6^; ∆H^o^ ~ 20 kcal/mol) (2, 25, 29). Also the fast reclosing allows solvent catalysis of the imino proton due to its EX2 nature. Multiple opening and closing reactions occur before its exchange is accomplished, allowing for interaction with solvent catalysts. In contrast, H-T exchange studies of DNA and a range of large polynucleotides find that base pair reclosing is far slower, ~10/s, leading to open states that are long-lived (ms) and to high open state population (Kop ~ 1%, ∆H^o^ ~ 5 kcal/mol). Imino proton exchange from the open state is faster than reclosing, directly indicating the fast opening-limited base pair opening rate (EX1) and indirectly the sizable opening equilibrium.

It has seemed obvious to all investigators that both of these clashing pictures could not be correct. NMR practitioners have offered differing interpretations although with all agreeing that the NMR results discredit the earlier H-T exchange interpretation (2). In fact, after some considerable uncertainty early on, a great deal of more recent work clearly shows that both H-T exchange and NMR methods provide credible and accurate HX results. This evaluation suggests a reason for the conflict between H-T and NMR observations that has not been considered before. However unlikely it may seem, I consider the proposition that both versions are correct. This hypothesis is supported by the widely different parameters obtained by the different methods when applied to the two different molecular models. Apparently, nucleotide polymers support two different modes of base pair opening with very different physical parameters. They are seen selectively by the different HX methods for reasons that become apparent when considered in this light.

### What Do the Base Pair Open States Look Like?

**Fig. 6.**
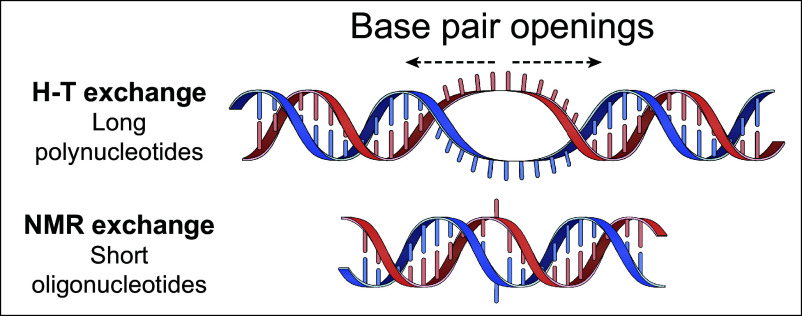
Evidence described in this paper indicates that both ribo and deoxyribo polymers harbor two modes of dynamic reversible base pair opening. HX studies of short oligonucleotides by NMR detect single base pair openings with fast reclosing rates (kcl ~10^6^/s) and catalyzable EX2 behavior. This mode loses two stacking interactions per opening, therefore has high enthalpy and very small equilibrium open occupation (Kop ~10^−5^). H-T exchange studies of long polynucleotides detect a more extensive open loop mode, perhaps 10 base pairs or more in size, which maintains stacking interactions and so has low enthalpy, low free energy, and high equilibrium occupation with Kop approaching 1% (Kop ~10^−2^). The open loop accounts for the measured base pair opening and closing rates by diffusing along the polynucleotide, mimicking soliton behavior. These two different kinds of openings are seen selectively in the different molecular models studied by different HX methods. In large polynucleotides, HX is dominated by highly populated extensive openings. Short oligonucleotides are unable to harbor such extensive open states. Their HX is dominated by less populated single base pair open states. The single base pair openings although undoubtedly also present in large polynucleotides have 1,000-fold lower occupation and so make no significant contribution to the HX measured for long polynucleotides.

The most striking characteristics of the NMR-determined reaction are the very fast reclosing rate (10^6^/s) and the low equilibrium open state occupation (10^–6^). The closing rate is close to the microsecond value found before for the rate of single base pair restacking (30, 31). The low occupation is consistent with the large value for unfavorable unstacking of a DNA base pair at temperature far below melting [∆H^o^ ~20 kcal/mol when both surfaces are unstacked (32)]. These observations suggest that the opening mode detected in short oligonucleotides by NMR represents the unstacking and swinging out of something close to a single base pair, as in Fig. 6.

Quite different parameters have been measured for polynucleotides (Figs. 2 and 3) including DNA (9, 17). Even though the large polynucleotides display high stability with melting temperature from 60 °C to well over 100 °C, base pair equilibrium opening constants are surprisingly large (Kop ~10^−2^), even at low temperature. Also, base pair reclosing rates (~10/s) are much slower than the microsecond rates known for single base pair restacking (30, 31). Measured thermodynamic parameters (∆G^o^ ~2 kcal/mol, ∆H^o^ ~5 kcal/mol), are much lower than literature values for thermal melting which is characterized by a larger unstacking enthalpy. The indication is that the open state that dominates polynucleotide HX involves multiple base pairs, yet largely maintains its base stacking (see Fig. 6). The same conclusion is supported by results for DNA (Fig. 5) (9, 17, 33).

These observations explain why the two different base pair opening modes are seen selectively by the different HX methods. Tritium exchange measured for long polynucleotides is dominated by fairly extensive multi-base pair opening reactions with a relatively large equilibrium open population. The small oligomers studied by NMR, under 15 base pairs in length, are too small to harbor the extensive base pair openings that determine the HX of large polymers. Accordingly, oligomer HX is dominated by a less populated class of openings. Its parameters are as expected for single nucleotide opening and closing. The same class of single nucleotide base pair openings undoubtedly occurs also in the large polynucleotide duplexes studied by H-T exchange, but they have much lower equilibrium occupation, with Kop ~10^−6^ compared to ~10^−2^ for the extensive openings that dominate measured H-T exchange (Table 1). Therefore, the minimally populated single nucleotide openings that determine oligonucleotide HX make no *perceptible* contribution to the HX measured for long polynucleotides.

### The Soliton Hypothesis.

The polynucleotide base pair opening parameters, wholly unexpected from past considerations, led me to a trapped migrating loop model, represented in Fig. 6 (13, 34). The hypothetical loop contains a number of base pair opened nucleotides, perhaps 10 or so. Stacking is maintained in the open state, as indicated by the low ∆H^o^ for opening (unstacking has high ∆H^o^). The open loop must be formed initially by several steps of longitudinal chain back untwisting so that a set of base pairs becomes unpaired. Repair of the twist defect would require a statistically unlikely set of retwisting moves, making the loop lifetime long. Instead, driven by thermal whole chain rotations, the open loop diffuses back and forth in a random walk along the duplex. New base pairs are opened and incorporated into the open loop at the advancing front, spend time within, and reclose upon exiting as either loop edge passes them. As a result the loop executes a diffusive search, exposing a changing stretch of open base pairs that could provide an initial docking site for interacting proteins and nucleic acids.

In the migrating loop picture, measured HX is determined by the rates for entering (kop) and exiting (kcl) the loop and the fractional population of base pairs within open loops (Kop) (Eqs. **1**–**4**). The base pair opening rate, about 1/s, is given by the immediate exchange (EX1) of the fast imino protons as base pairs first open upon loop entry. For the amino protons with slower HX chemistry, an initial opening does not lead to immediate exchange except in the presence of strong catalysis. The base pair equilibrium opening constant, Kop, calculated from the HX protection factor, commonly leads to a Keq value on the order of 0.01, suggesting that about 1% of base pairs occupy open loops (Table 1). These values also yield the apparent reclosing rate, kop/Kop ~20/s, which suggests that diffusive soliton travel acts to reclose about half its open base pairs, i.e., to experience ~20 Å of displacement (five base pairs), in about 50 ms.

The original traveling wave idea (13) was elaborated in a sophisticated analysis in roundtable discussions with half of our physics department including two Nobelists (Schrieffer, Heeger) (34). They realized that the traveling loop idea mirrored a well-known (to them) species of long-lived wave packets known as solitons (35, 36), encountered in many contexts in the world of physics (optics, magnetics, hydrodynamics) but not yet in biologically interesting molecules. In mathematics and physics (quoting Wikipedia), a soliton is a nonlinear, self-reinforcing, localized wave packet that is strongly stable and preserves its shape while propagating freely, at constant velocity, due to a balanced cancellation of nonlinear and dispersive effects (36). In spite of a large literature that has since been devoted to traveling loops or bubbles in DNA, almost all of a theoretical nature, and the DNA-soliton concept with currently over 700 citations (34), my impression is that the soliton hypothesis is still unproven. Some circumstantial evidence comes from the absence of solitonic behavior in the small oligonucleotides studied by NMR HX, despite the fact that they dominate larger polynucleotide HX. The short length of oligonucleotides rules out extended solitons.

A part of the theoretical literature has challenged the soliton hypothesis based on the picture that considerations of solvent viscosity and soliton inertia rule out the ability of a soliton wave to transport material along the length of the duplex. Actually, DNA solitons do not translocate material. Unlike solitonic water waves, it is only the open void rather than material nucleotides that appears to travel along the DNA length. The base pairs remain in place and just open and close individually as usual at the leading and trailing edges.

The current state of theoretical analysis suggests that the possible reality of multi-base pair soliton-like loops will have to be sought experimentally. I think this might be done in short order by cryoelectron microscopy.

## Summary

In summary, contrary to current belief, the apparently contradictory versions of DNA dynamics derived from different kinds of HX experimentation are both correct in their own way. The results organized here indicate that two different modes of base pair opening with very different structural and dynamical parameters are seen selectively in small and large polynucleotides. A single nucleotide opening mode is isolated for observation in small oligonucleotides and has been much studied (37). Given its restricted reach and minimal occupation, this opening mode seems unlikely to provide useful information on DNA structural dynamics and interactions. The multi-base pair opening mode detected in DNA and large polynucleotides seems more likely to do so. In these studies, the special character of soliton dynamics may play an important role.

## Methods

### H-T Exchange.

In the H-T exchange-gel filtration method (1), the polynucleotide is placed in a solution of any desired composition and condition and a small aliquot of tritiated water (THO) is added to initiate H to T exchange. Timed samples are taken, the tritium labeled polymer is separated from tritiated solvent by fast gel filtration (~1 min), and the remaining polynucleotide-bound tritium is measured by liquid scintillation counting. A series of timed samples shows the HX time course. The method has proven to be precise, artifact-free, and exceptionally revealing over decades of work with proteins and nucleic acids (11).

### H-H Exchange by NMR.

The early H-T exchange work motivated parallel HX studies by NMR methods. NMR provides the advantage of being able to identify and measure the HX of individual nucleotides. However, this capability was limited to short rapidly tumbling oligonucleotides less than about 15 base pairs in length and to the imino protons which resonate far downfield, around −13 ppm. Amino proton resonances are at even lower field, generally out of reach.

There were difficulties with the NMR measurement. Imino proton exchange is too fast to be measured by the direct replacement of exchangeable protons in D_2_O, so the field turned to the measurement of HX-dependent T1 relaxation by inversion recovery (38) or T2 relaxation measured by line broadening (24). However, the measured relaxation has a major background component due to non-HX magnetic dipolar effects (23). The field has learned to extract the component of NMR relaxation due to HX reactions alone by virtue of its dependence on temperature, but this requirement is limiting. The problem can be largely avoided by focusing the NMR measurement on solvent water rather than the polymer itself, measuring the HX-dependent spin lattice T1 relaxation of the water solvent by inversion recovery, as in Fig. 4 (19, 21, 22).

## Data Availability

Study data are included in the main text.
